# Van Maldergem syndrome-1 in a patient with central precocious puberty: A case report

**DOI:** 10.1097/MD.0000000000043550

**Published:** 2025-08-08

**Authors:** Yeping Wang, Lingjing Ying, Yuxuan Dai, Xiaoyun Jiang, Zubi Liu, Kaixuan Wang, Bo Xu

**Affiliations:** a Pediatric Department , Jinhua Municipal Central Hospital, Jinhua, China; b Pediatric Department , Jinhua Maternity and Child Health Care Hospital, Jinhua, China; c Key Laboratory of Digital Technology in Medical Diagnostics of Zhejiang Province, Dian Diagnostics Group Co., Ltd., Hangzhou, China; d Thoracic Surgery Department, Jinhua Municipal Central Hospital, Jinhua, China.

**Keywords:** case report, craniofacial deformity, DCHS1, intellectual disability, Van Maldergem syndrome

## Abstract

**Rationale:**

Van Maldergem syndrome-1 (VMS-1; OMIM #601390) is a multisystem genetic disease characterized by intellectual disability, craniofacial deformities, skeletal anomalies, and/or other variable malformations. Few cases have been reported to date, posing challenges to the diagnosis and management of this condition.

**Patient concerns:**

A 7-year-old female Chinese patient presented with a series of developmental defects, including precocious puberty, mild intellectual disability, unusual craniofacial features, mild shortening of the fourth metacarpal bone, and clumsy movements with poor coordination.

**Diagnoses:**

The patient was diagnosed with central precocious puberty and VMS-1 based on clinical symptoms and genetic results.

**Interventions:**

The treatment for precocious puberty involved monthly administration of leuprorelin acetate microspheres (Enantone^®^). Neurodevelopmental deficits were managed with regular follow-ups due to the lack of established therapeutic protocols.

**Outcomes:**

Over the 2-year follow-up, the precocious pubertal development was successfully controlled, and the neurodevelopmental deficits remained stable without progression.

**Lessons:**

This case highlights the hallmark clinical features of VMS-1, including neurodevelopmental impairment and craniofacial anomalies, while also expanding the known genetic spectrum of the disorder. These findings provide valuable insights into the diagnosis and management of this extremely rare genetic condition.

## 1. Introduction

Van Maldergem syndrome (VMS) is an extremely rare autosomal recessive disorder characterized by intellectual disability (ID), distinctive craniofacial features, hearing impairment, skeletal and limb anomalies, brain abnormalities (including periventricular nodular heterotopia), and/or other variable malformations.^[1,2]^ VMS is classified into 2 subtypes based on its molecular etiology. VMS-1 (OMIM #601390) results from homozygous mutations in the gene encoding Dachsous cadherin-related 1 (*DCHS1*) located on chromosome 11p15.4, while VMS-2 (OMIM #615546) is caused by biallelic variants in the gene encoding FAT atypical cadherin 4 (*FAT4*) on chromosome 4q28.1.^[1]^ Despite their distinct genetic causes, both subtypes exhibit similar clinical phenotypes.

To date, only 17 VMS cases have been reported worldwide.^[1–10]^ Limited data and the absence of a standardized treatment protocol provide further challenges to the diagnosis and management of this ultrarare condition. Here, we present a case of concurrence of precocious puberty and VMS-1.

## 2. Case report

**Figure 1.**
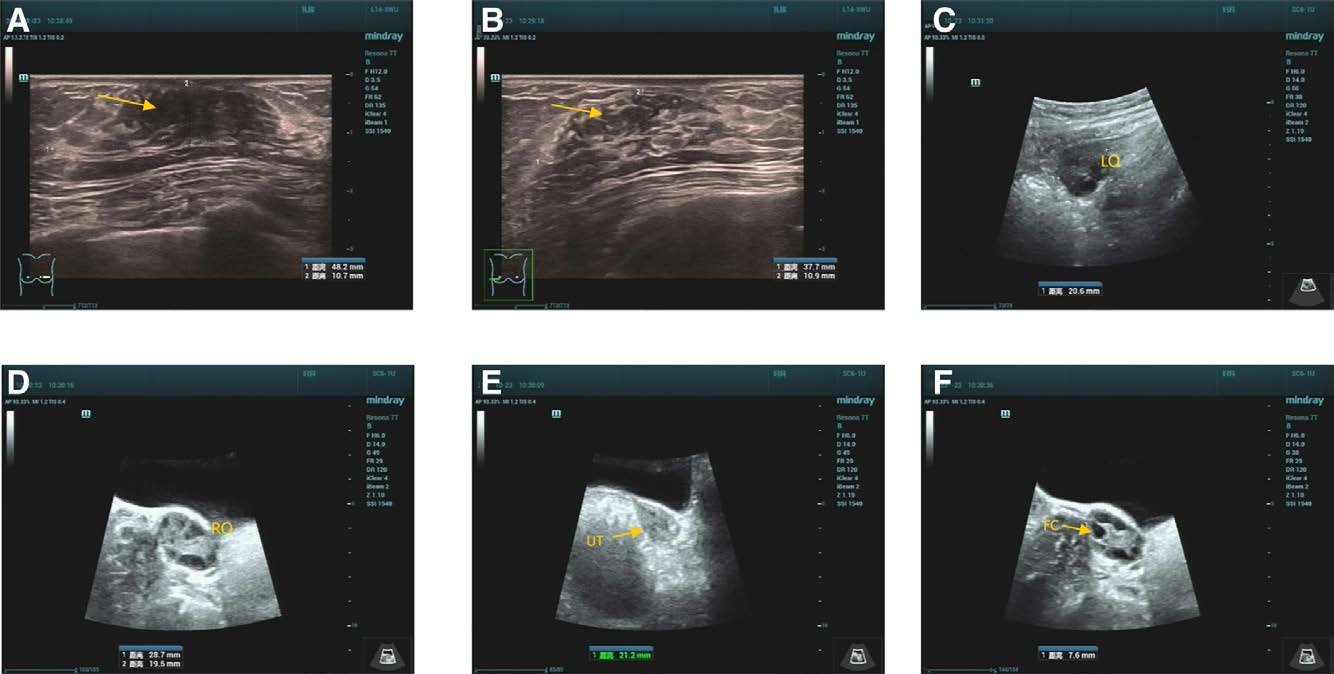
Ultrasound scans on the breasts and reproductive organs. (A) Left breast (size: 48.2 mm × 10.7 mm); (B) right breast (size: 37.7 mm × 10.9 mm), yellow arrows in (A, B) indicate the mammary glands; (C) left ovary (LO, size: 30.8 mm × 17.3 mm × 20.6 mm); (D) right ovary (RO, size: 28.7 mm × 19.5 mm × 25.9 mm); (E) uterus (UT, length: 46 mm); (F) follicles (FC). FC = follicles, LO = left ovary, RO = right ovary, UT = uterus.

In 2021, a Chinese girl, aged 7 years and 10 months and born to healthy nonconsanguineous parents, was referred to our clinic for breast development persisting over 1 year. Her family history revealed no obvious abnormalities. The patients height (132.6 cm) and weight (37 kg) were both above the 90th percentile. Bone age assessment indicated an advanced skeletal age of 11 years. Breast development was at Tanner stage 4, and pubic hair was present. Laboratory tests revealed a basal follicle-stimulating hormone level of 3.05 mIU/mL and a basal luteinizing hormone (LH) level of 0.62 mIU/mL. The peak serum LH value was 5.6 mIU/mL, while the peak LH/follicle-stimulating hormone ratio after stimulation with gonadotropin-releasing hormone exceeded 0.6, indicative of early activation of the hypothalamic-pituitary-gonadal axis. The level of serum estradiol was 51 pg/mL, and that of insulin-like growth factor 1 was elevated at 400 ng/mL. The results of thyroid function tests were within normal limits. Ultrasound scans showed increases in the sizes of the bilateral mammary glands, ovaries (with multiple follicles >4 mm in diameter), and the uterus relative to age peers (Fig. 1). Thus, the patient exhibited the typical characteristics of central precocious puberty (CPP).^[11]^

**Figure 2.**
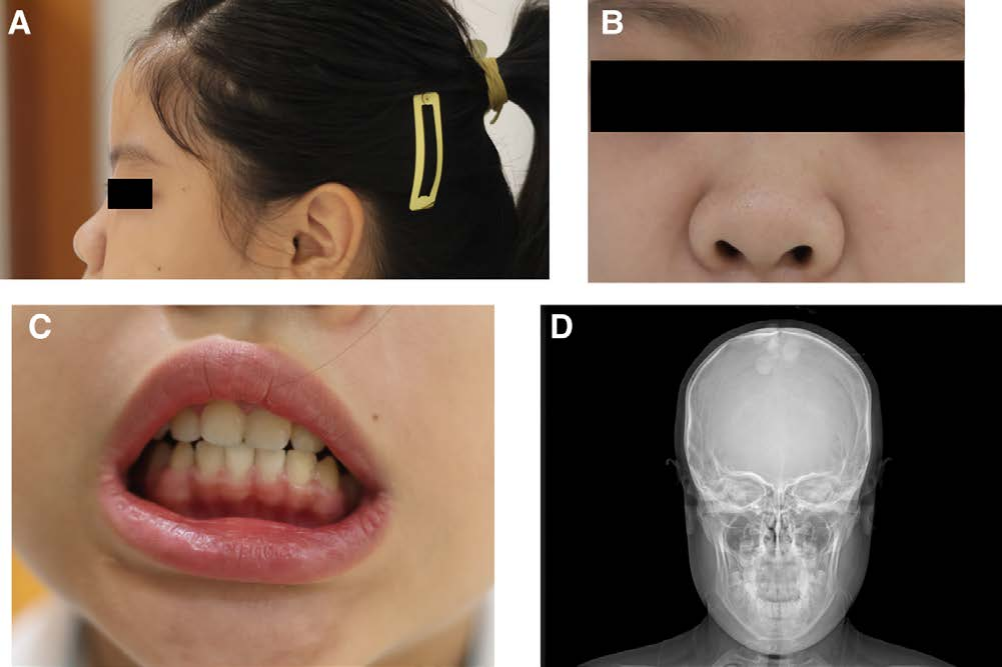
Craniofacial features of the patient. (A–C) Distinctive facial appearance, characterized by flat forehead, hypertelorism, epicanthal folds, gingival enlargement, thick nasal wings, flat nasal tip, and a flat and broad nasal bridge. Black bars covering the eyes in (A, B) are used to protect the patient privacy. (D) X-ray scan showing micrognathia, maxillary hypoplasia, and slightly widened cranial suture.

Additionally, distinctive facial features were observed in the patient. These included a flat facial profile, flattened forehead, long philtrum, hypertelorism, epicanthal folds, narrow palpebral fissures, a large mouth, everted lower lip, gingival hypertrophy, irregular dentition, a small mandible, and characteristic nasal features (thickened alae nasi, a broad and flat nasal bridge, and a rounded, bulbous nasal tip; Fig. 2A–C). Radiographic examination of the skull showed micrognathia, maxillary hypoplasia, and slightly widened cranial sutures (Fig. 2D). Radiographs of the hands revealed mild shortening of the fourth metacarpal bone.

Developmentally, the patient exhibited delays in both language and motor milestones compared to peers. She walked at the age of 18 months, and displayed clumsy movements with poor coordination, with frequent falls. At her first visit, she showed monotonic speech, intellectual delays, and moderate learning difficulties. Neurocognitive testing revealed mental retardation with an IQ of 58, consistent with mild ID. Auditory examinations were normal. Renal ultrasound scans revealed normal kidney size and morphology, though small kidney stones were detected. No lymphatic anomalies were observed.

**Figure 3.**
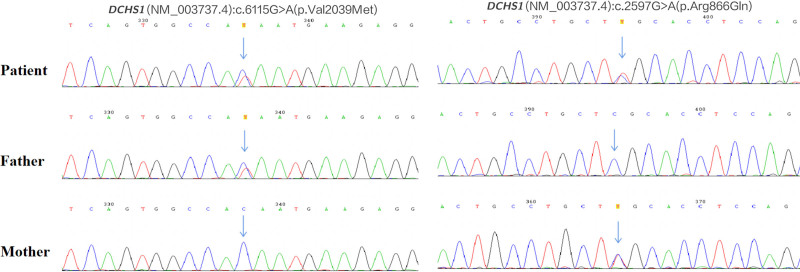
Sanger sequencing verification of the 2 *DCHS1* variants in the family.

Given the combination of mild ID and distinctive craniofacial features, a genetic etiology was suspected. Peripheral blood was collected for karyotype analysis, chromosomal microarray, and whole-exome sequencing. No chromosomal abnormalities were detected (46, XX). However, whole-exome sequencing identified compound heterozygous missense variants in the *DCHS1* gene, NM_003737.4 (*DCHS1*): c.6115G > A (p.Val2039Met) and c.2597G > A (p.Arg866Gln). Sanger sequencing confirmed the presence of these *DCHS1* variants and their segregation within the family (Fig. 3). Both variants are listed as heterozygous variants in the gnomAD database, although they are very rare (Table 1). Neither variant has been reported in a homozygous or compound heterozygous state in any database or study to our knowledge. In silico tools (CADD, SIFT, and PolyPhen-2) were used to predict pathogenicity (Table 1). CADD and PolyPhen-2 described both variants as disease-causing, while SIFT classified the p.Arg866Gln variant as tolerated. No additional pathogenic or likely pathogenic variants were identified. According to the guidelines of the American College of Medical Genetics and Genomics,^[12]^ we classified the 2 variants as likely pathogenic. Considering the genetic findings and clinical presentation, the patient was also diagnosed with VMS-1.

**Table 1:** Genetic analysis of the 2 *DCHS1* variants in the patient.

Nucleotide variant	c.6115G > A	c.2597G > A
Amino acid change	p.Val2039Met	p.Arg866Gln
Origin of inheritance	Father	Mother
Allele frequency in gnomeAD v4.1.0	6.843E−07	3.49E−05
Number of homozygotes in gnomeAD v4.1.0	0	0
CADD score*	23.2	22.4
SIFT prediction	Deleterious	Tolerated
PolyPhen-2 prediction	Probably damaging	Probably damaging

CADD = combined annotation dependent depletion, PolyPhen-2 = polymorphism phenotyping v2, SIFT = sorting intolerant from tolerant.

* In CADD prediction, the score >20 indicates the deleteriousness of a single nucleotide variant.

For the treatment of CPP, leuprorelin acetate microspheres (Enantone^®^) at a monthly dose of 3.75 mg were administered. At the most recent follow-up visit, when the patient was aged 10 years and 4 months, her bone age was assessed at 11.5 years, with a height of 147.7 cm (75th centile) and a weight of 38.53 kg (75th centile). Furthermore, all laboratory test results were within normal ranges, suggesting that CPP was well managed with Enantone^®^.

In terms of VMS-1, due to the absence of a standardized treatment protocol and the relatively mild severity of the disease, no additional interventions were administered. By the age of 10, the patient continued to present with motor incoordination, speech impairment, and learning difficulties, suggesting that the severity of ID remained largely unchanged.

## 3. Discussion

This report describes a female Chinese patient with concurrent CPP and VMS-1, diagnosed through a combination of clinical evaluation and molecular genetic analyses. The key clinical features supporting the diagnosis of VMS-1 included ID, atypical facial appearance, micrognathia, and maxillary hypoplasia, all of which are consistent with findings reported in previous studies.^[1–10]^ However, unlike most previously reported patients with VMS, who often display additional abnormalities involving the ears, limbs, trachea, or brain, the present patient exhibited only a mild shortening of the fourth metacarpal bone. Her neurodevelopmental condition remained stable during follow-up, suggesting relatively mild disease severity, which justified the absence of pharmacological or surgical intervention.

The main difference between the present case and previously reported patients with VMS is the occurrence of CPP, a condition characterized by early secondary sexual development, accelerated growth velocity, and advanced bone age.^[13]^ CPP is notably prevalent in Chinese girls (~11.47%) and can complicate the recognition of underlying neurodevelopmental disorders.^[14]^ Unfortunately, we have insufficient evidence on the relationship between CPP and VMS. Indeed, most patients with VMS exhibit growth retardation rather than growth acceleration, and a previous study reported a VMS case with endocrine abnormalities, hypogonadotropic hypogonadism, and breast hypoplasia.^[9]^ Therefore, we speculate that the CPP in this case was independent of VMS.

VMS was first described in 1992 by Van Maldergem et al but its genetic basis was not elucidated until 2013 when Cappello et al identified biallelic variants in *DCHS1* and *FAT4* in 9 VMS patients from 7 unrelated families.^[1,2]^ Since then, additional VMS cases with biallelic *DCHS1* or *FAT4* variants have been reported. In the present case, the patient carried novel compound heterozygous missense variants in *DCHS1*: p.Val2039Met and p.Arg866Gln. While both variants are present in the general population in a heterozygous state (albeit rarely), their biallelic occurrence (homozygous or compound heterozygous) has not been previously reported. This study provides case-level evidence supporting the pathogenicity of these variants, although functional studies are needed to elucidate their specific effects on the function of the DCHS1 protein.

The molecular mechanisms by which variants in *DCHS1* and *FAT4* cause VMS are not yet fully understood. The DCHS1-FAT4 receptor-ligand pair plays a critical role in mammalian neurogenesis, neuronal migration, and cortical architecture, which may explain the brain malformations (e.g., dysmorphic corpus callosum, bilateral nodular periventricular heterotopias) observed in some VMS patients.^[1,15,16]^ Furthermore, animal studies have suggested that DCHS1-FAT4 signaling is essential for osteoblast differentiation, potentially explaining the craniofacial abnormalities characteristic of VMS.^[17]^ Notably, both DCHS1 and FAT4 also function independently. For example, DCHS1 has been reported to regulate the microtubule cytoskeleton during early embryogenesis independent of FAT4, while FAT4 interacts with the RET signaling pathway during kidney development.^[18,19]^

The limitations of this study include: we did not perform functional experiments to validate the pathogenicity of the *DCHS1* variants; there is insufficient evidence to support a causal relationship between VMS-1 and CPP in this patient; given the limited follow-up period of 2 years, the patient’s long-term progression remains to be determined; the severity of ID has shown no marked improvement due to the absence of a standardized treatment protocol.

In summary, this case report describes the coexistence of VMS-1 and CPP in a Chinese patient with novel compound heterozygous *DCHS1* variants. Despite the highly variable phenotypic spectrum of VMS, neurodevelopmental defects and craniofacial deformities remain its most characteristic features. This study reinforces the understanding of VMS, reminding clinicians of this ultra-rare genetic condition in clinical practice, especially when the patient displays growth or developmental abnormalities.

## Author contributions

**Conceptualization:** Yeping Wang, Kaixuan Wang, Bo Xu.

**Data curation:** Yeping Wang, Lingjing Ying, Yuxuan Dai.

**Formal analysis:** Yuxuan Dai, Xiaoyun Jiang, Zubi Liu.

**Funding acquisition:** Yeping Wang.

**Investigation:** Lingjing Ying, Yuxuan Dai.

**Methodology:** Lingjing Ying.

**Project administration:** Yeping Wang.

**Resources:** Lingjing Ying, Zubi Liu.

**Supervision:** Bo Xu.

**Validation:** Zubi Liu, Kaixuan Wang.

**Writing – original draft:** Yeping Wang.

**Writing – review & editing:** Kaixuan Wang, Bo Xu.

## References

[1] Cappello S Gray MJ Badouel C. Mutations in genes encoding the cadherin receptor-ligand pair DCHS1 and FAT4 disrupt cerebral cortical development. Nat Genet. 2013;45:1300–8. 24056717 10.1038/ng.2765

[2] Mansour S Swinkels M Terhal PA. Van Maldergem syndrome: further characterisation and evidence for neuronal migration abnormalities and autosomal recessive inheritance. Eur J Hum Genet. 2012;20:1024–31. 22473091 10.1038/ejhg.2012.57 PMC3449074

[3] Ivanovski I Akbaroghli S Pollazzon M. Van Maldergem syndrome and Hennekam syndrome: further delineation of allelic phenotypes. Am J Med Genet A. 2018;176:1166–74. 29681106 10.1002/ajmg.a.38652

[4] de Meij TGJ Zwijnenburg PJG Broers CJM Bökenkamp A. Intestinal lymphangiectasia-a novel finding in Van Maldergem syndrome challenging the role of lymphedema for the distinction from Hennekam syndrome. Am J Med Genet A. 2019;179:1398–9. 31063239 10.1002/ajmg.a.61178

[5] Neuhann TM Müller D Hackmann K Holzinger S Schrock E Di Donato N. A further patient with Van Maldergem syndrome. Eur J Med Genet. 2012;55:423–8. 22469822 10.1016/j.ejmg.2012.02.012

[6] Verheij E Thomeer HG Pameijer FA Topsakal V. Middle ear abnormalities in Van Maldergem syndrome. Am J Med Genet A. 2017;173:239–44. 27739185 10.1002/ajmg.a.37990

[7] Ulubas Isik D Unal S Erol S Arslan Z Bas AY Demirel N. A newborn diagnosed with Van Maldergem syndrome. Clin Dysmorphol. 2018;27:63–5. 29505454 10.1097/MCD.0000000000000211

[8] Rahmani N Ahmadvand M Khakpour G. Use of expanded carrier screening for retrospective diagnosis of two deceased siblings with Van Maldergem syndrome 2: case report. Asian Biomed (Res Rev News). 2023;16:322–8. 37551355 10.2478/abm-2022-0036 PMC10392142

[9] Sotos J Miller K Corsmeier D. A patient with Van Maldergem syndrome with endocrine abnormalities, hypogonadotropic hypogonadism, and breast aplasia/hypoplasia. Int J Pediatr Endocrinol. 2017;2017:12. 29046692 10.1186/s13633-017-0052-z PMC5640965

[10] Alaqeel B Babiker A Al Mutairi F Al Dubayee M. Coexistence of genetic conditions: exploring a possible relationship. Sudan J Paediatr. 2019;19:60–6. 31384091 10.24911/SJP.106-1554459680 PMC6589797

[11] The Subspecialty Group of Endocrinologic, Hereditary and Metabolic Diseases, the Society of Pediatrics, Chinese Medical Association; Editorial Board, Chinese Journal of Pediatrics. Expert consensus on the diagnosis and treatment of central precocious puberty (2022) [in Chinese]. Zhonghua Er Ke Za Zhi . 2023;61:16–22. 36594116 10.3760/cma.j.cn112140-20220802-00693

[12] Richards S Aziz N Bale S.; ACMG Laboratory Quality Assurance Committee. Standards and guidelines for the interpretation of sequence variants: a joint consensus recommendation of the American College of Medical Genetics and Genomics and the Association for Molecular Pathology. Genet Med. 2015;17:405–24. 25741868 10.1038/gim.2015.30 PMC4544753

[13] Zevin EL Eugster EA. Central precocious puberty: a review of diagnosis, treatment, and outcomes. Lancet Child Adolesc Health. 2023;7:886–96. 37973253 10.1016/S2352-4642(23)00237-7

[14] Liu Y Yu T Li X. Prevalence of precocious puberty among Chinese children: a school population-based study. Endocrine. 2021;72:573–81. 33528762 10.1007/s12020-021-02630-3

[15] Zakaria S Mao Y Kuta A. Regulation of neuronal migration by DCHS1-FAT4 planar cell polarity. Curr Biol. 2014;24:1620–7. 24998526 10.1016/j.cub.2014.05.067 PMC4193925

[16] Beste C Ocklenburg S von der Hagen M Di Donato N. Mammalian cadherins DCHS1-FAT4 affect functional cerebral architecture. Brain Struct Funct. 2016;221:2487–91. 25930014 10.1007/s00429-015-1051-6

[17] Crespo-Enriquez I Hodgson T Zakaria S. DCHS1-FAT4 regulation of osteogenic differentiation in mouse. Development. 2019;146:dev176776. 31358536 10.1242/dev.176776 PMC7376788

[18] Li-Villarreal N Forbes MM Loza AJ. Dachsous1b cadherin regulates actin and microtubule cytoskeleton during early zebrafish embryogenesis. Development. 2015;142:2704–18. 26160902 10.1242/dev.119800 PMC4529026

[19] Zhang H Bagherie-Lachidan M Badouel C. FAT4 fine-tunes kidney development by regulating RET signaling. Dev Cell. 2019;48:780–92.e4. 30853441 10.1016/j.devcel.2019.02.004 PMC6766079

